# Heterogeneous Integration of Wide Bandgap Semiconductors and 2D Materials: Processes, Applications, and Perspectives

**DOI:** 10.1002/adma.202411108

**Published:** 2024-10-19

**Authors:** Soo Ho Choi, Yongsung Kim, Il Jeon, Hyunseok Kim

**Affiliations:** ^1^ Department of Nano Engineering Department of Nano Science and Technology SKKU Advanced Institute of Nanotechnology (SAINT) Sungkyunkwan University (SKKU) Suwon 16419 Republic of Korea; ^2^ Department of Electrical and Computer Engineering Nick Holonyak, Jr. Micro and Nanotechnology Laboratory University of Illinois Urbana‐Champaign Urbana IL 61801 USA; ^3^ Department of Materials Science and Engineering Nick Holonyak, Jr. Micro and Nanotechnology Laboratory University of Illinois Urbana‐Champaign Urbana IL 61801 USA

**Keywords:** 2D materials, applications, fabrication, heterostructure, wide‐bandgap semiconductors

## Abstract

Wide‐bandgap semiconductors (WBGs) are crucial building blocks of many modern electronic devices. However, there is significant room for improving the crystal quality, available choice of materials/heterostructures, scalability, and cost‐effectiveness of WBGs. In this regard, utilizing layered 2D materials in conjunction with WBG is emerging as a promising solution. This review presents recent advancements in the integration of WBGs and 2D materials, including fabrication techniques, mechanisms, devices, and novel functionalities. The properties of various WBGs and 2D materials, their integration techniques including epitaxial and nonepitaxial growth methods as well as transfer techniques, along with their advantages and challenges, are discussed. Additionally, devices and applications based on the WBG/2D heterostructures are introduced. Distinctive advantages of merging 2D materials with WBGs are described in detail, along with perspectives on strategies to overcome current challenges and unlock the unexplored potential of WBG/2D heterostructures.

## Graphical abstract

The heterogeneous integration of wide‐bandgap semiconductors (WBGs) and 2D materials is emerging as a promising way to address various challenges faced by WBGs. This review covers recent advancements in fabrication techniques, mechanisms, devices, and novel functionalities of WBG/2D heterostructures. Furthermore, the directions and perspectives are outlined for realizing practical applications in the near future.

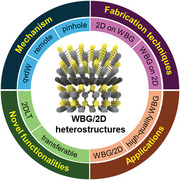

## 1 Introduction

Wide‐bandgap semiconductors (WBGs) have garnered significant interest over the last few decades owing to their exceptional electrical, optical, and thermal properties, which make them suitable for applications in which conventional semiconductors fall short. WBGs typically refer to materials with bandgaps greater than 2 eV that can be doped to facilitate carrier transport; notable examples include silicon carbide (SiC) and gallium nitride (GaN).^[^ ^1^ ^]^ High electron saturation velocities and thermal stabilities of these materials make them ideal for diverse high‐power electronic applications.^[^ ^2^, ^3^, ^4^ ^]^ Furthermore, direct‐bandgap WBGs are utilized in optoelectronic and photonic applications across visible and ultraviolet (VIS/UV) wavelengths.^[^ ^5^, ^6^, ^7^, ^8^, ^9^, ^10^, ^11^, ^12^ ^]^ Recently, WBGs have attracted considerable attention owing to their potential in electric vehicles, power grids, 5G communications, and quantum applications. To further enhance the performance and expand the operation regime, novel ultrawide‐bandgap semiconductors (UWBGs) with even larger bandgaps are emerging, including materials such as aluminum nitride (AlN), gallium oxide (Ga_2_O_3_), boron nitride, and diamond, some of which were previously considered dielectrics rather than semiconductors.^[^ ^13^, ^14^ ^]^

However, unlike conventional semiconductors such as silicon, WBGs and UWBGs cannot be easily fabricated into high‐quality wafers using the Czochralski method, except for β‐Ga_2_O_3_ substrates. Consequently, WBG and UWBG substrates are expensive and typically available only for small diameters. The scarcity of suitable growth substrates poses a significant bottleneck in the formation of high‐quality device layers. For instance, GaN or AlN device layers are often grown on sapphire, SiC, or Si substrates, where lattice and thermal mismatches between the epitaxial layers and substrates induce high dislocation densities, degrading the device performance.^[^ ^15^, ^16^, ^17^, ^18^, ^19^, ^20^, ^21^, ^22^, ^23^ ^]^ In addition, the wide bandgaps and associated work functions of these materials make it challenging to achieve reliable metal contact or doping. Fundamental breakthroughs are essential to overcome these obstacles and push the boundaries of material properties, device performance, and functionality of WBG devices.

**Figure 1:**
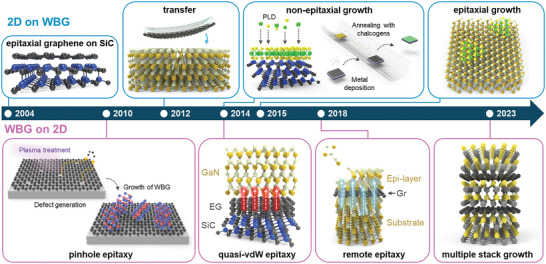
Evolution of fabrication techniques for WBG/2D heterostructures. Upper panel: Formation of 2D materials on WBGs. Bottom panel: Formation of WBGs on 2D materials.

In this context, the utilization of layered 2D materials in conjunction with WBG is emerging as a promising solution. Integrating these materials, whether WBG‐on‐2D or 2D‐on‐WBG, offers new degrees of freedom in material engineering and device design. First, 2D materials can serve as epitaxial templates for the growth of high‐quality WBGs, significantly enhancing their crystallinity and benefiting a wide array of WBG devices.^[^ ^24^, ^25^ ^]^ Second, 2D materials can be employed as functional layers within WBG device platforms, acting as carrier transport layers and heat spreaders.^[^ ^26^, ^27^ ^]^ The unique chemical, electrical, mechanical, and optical properties of sp^2^‐bonded 2D materials make them ideal for these new functionalities. Third, 2D materials facilitate the transfer and heterogeneous integration of WBGs, paving the way for exploring new physical phenomena and applications.^[^ ^28^, ^29^, ^30^ ^]^ This integration has sparked significant interest among researchers, leading to remarkable progress in recent years, as summarized chronologically in **Figure** **1**.

In this review, we present the recent advances in the integration of WBGs and 2D materials. Given the increasing significance of WBGs and the promising roles of 2D materials in WBG devices, it is an opportune time to summarize the various fabrication techniques and their applications. We aim to provide a comprehensive overview and perspective on the fabrication techniques, mechanisms, and devices based on WBG/2D heterostructures. First, we briefly describe the properties of representative WBGs and 2D materials. Next, we introduce various methods for integrating these two distinct material families, such as the formation of WBGs on 2D materials and vice versa, using both epitaxial and nonepitaxial methods, and discuss associated current challenges and potential solutions. Finally, we highlight the devices and applications uniquely enabled by integrating WBGs with 2D materials and share our perspectives for future research.

## 2 Properties of Layered 2D Materials and Wide Bandgap Semiconductors

2D materials exhibit a layered structure with a typical layer thickness of 1 nm or less. Elements constituting a layer form sp^2^ bonds with each other, whereas stacked layers are bound by weak van der Waals (vdW) interactions rather than stronger covalent or ionic bonds.^[^ ^31^ ^]^ Such vdW interactions make 2D materials particularly interesting for integration with WBGs, as discussed below. Their ultrathin nature induces a quantum confinement effect, leading to the emergence of novel physical phenomena.^[^ ^32^, ^33^, ^34^ ^]^

**Figure 2:**
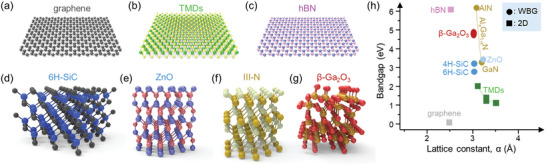
Crystal structure and bandgap of representative 2D materials and WBGs. a–g) Schematic illustration of the crystal structure of a) graphene, b) TMDs, c) hBN, d) 6H‐SiC, e) ZnO, f) III‐nitrides, and g) β‐Ga_2_O_3_. h) Diagram showing bandgaps and lattice constants of 2D materials and WBGs.

Among various 2D materials, graphene, transition metal dichalcogenides (TMDs), and hexagonal boron nitride (hBN) stand out as foremost representatives. These materials have been extensively investigated, earning recognition for their distinctive properties and potential applications. Graphene, composed of a monolayer of carbon atoms arranged in a hexagonal lattice (**Figure** **2**a), exhibits remarkable mechanical strength (≈130 GPa) and exceptional carrier mobility (>10^4^ cm^2^ V^−1^ s^−1^ at room temperature) positioning it as a promising material for transparent flexible electronics.^[^ ^35^, ^36^, ^37^ ^]^ Moreover, the high thermal conductivity of 3000–5000 W m^−1^ K^−1^ makes it ideal for thermal management applications.^[^ ^38^ ^]^ TMDs have a layered structure comprising alternating transition metal and chalcogen atom layers (Figure 2b). Depending on their elemental and crystal structures, TMDs exhibit metallic or semiconducting properties.^[^ ^39^ ^]^ This characteristic, coupled with the ability to manipulate the crystal structure and electronic properties, has garnered significant interest in TMDs for electronic device applications.^[^ ^40^, ^41^, ^42^, ^43^ ^]^ hBN, featuring a hexagonal lattice structure in which boron and nitrogen atoms alternate (Figure 2c), has an ultrawide bandgap of ∼6 eV, and exhibits insulating properties with exceptional stability and optical transparency.^[^ ^44^, ^45^ ^]^ Therefore, hBN can be used as an insulator in electronic devices and multilayer structures.^[^ ^46^, ^47^, ^48^ ^]^ Research exploring the potential applications of 2D materials across various fields is actively pursued, with significant prospects for their integration into real‐world products.

WBGs typically denote materials with bandgaps above ≈2 eV, larger than those of conventional semiconductors such as Si (≈1.12 eV), Ge (≈0.66 eV), or GaAs (≈1.42 eV at 300 K).^[^ ^1^ ^]^ Notable examples include zinc oxide (ZnO), SiC, Ga_2_O_3_, and III‐N compound semiconductors. WBGs are distinguished by their high electrical resistance to breakdown and stability at high temperatures, making them ideal for high‐power electronic devices as well as high‐frequency and high‐temperature applications.^[^ ^2^, ^3^, ^4^ ^]^ Among the several polytypes of SiC, wide‐bandgap hexagonal SiC is commonly categorized as 4H‐SiC or 6H‐SiC (Figure 2d) based on its stacking sequence with a bandgap ranging from 2.9 to 3.2 eV. Owing to its excellent thermal conductivity and chemical stability, hexagonal SiC is utilized in applications requiring stable operation in high‐temperature and high‐power environments, such as automotive electronic systems and high‐power electronic devices.^[^ ^49^ ^]^ ZnO predominantly features a wurtzite structure (Figure 2e) and possesses a wide bandgap (≈3.4 eV), along with high transparency and piezoelectricity,^[^ ^50^, ^51^, ^52^ ^]^ making ZnO suitable for applications in optoelectronic devices, ultraviolet (UV) light‐emitting diodes (LEDs), and sensors.^[^ ^53^, ^54^, ^55^, ^56^, ^57^ ^]^ ZnO has demonstrated outstanding performance in these areas, particularly in transparent electronic devices and optical sensors. III‐N compound semiconductors are composed of Group III elements such as gallium (Ga), aluminum (Al), and indium (In) paired with nitrogen (N) and typically exhibit a wurtzite structure (Figure 2g). The bandgap of these materials can be tailored based on their constituent elements, endowing them with exceptional electrical and optical properties.^[^ ^58^ ^]^ Consequently, III‐N compound semiconductors are suitable for application in high‐power electronic devices, and inorganic LEDs.^[^ ^59^, ^60^, ^61^ ^]^

Recently, UWBGs have emerged to improve device performance and operation regimes. Among various phases of Ga_2_O_3_, β‐Ga_2_O_3_ is the most stable and notable semiconductor material, featuring a wide bandgap of 4.6–5.0 eV, a monoclinic structure (Figure 2f), and excellent electrical properties and stability.^[^ ^62^, ^63^, ^64^ ^]^ This makes it particularly attractive for high‐power electronic device applications beyond GaN and SiC. Diamond and cubic boron nitride (c‐BN) have emerged as UWBGs. Diamond has a wide bandgap (5.5 eV), a carrier mobility exceeding 4000 cm^2^ V^−1^ s^−1^, and an impressive thermal conductivity of 20 W cm^−1^ K^−1^, rendering it an exceedingly promising material.^[^ ^65^, ^66^, ^67^ ^]^ However, its synthesis requires extremely high temperatures and challenging processing, owing to its exceptional hardness. c‐BN exhibits an ultrawide bandgap of 6.4 eV and the highest breakdown field among the WBGs.^[^ ^68^, ^69^ ^]^ Nonetheless, device applications utilizing c‐BN have rarely been reported due to the limited quality of synthetic c‐BN, influenced by its metastable nature under ambient conditions.^[^ ^69^ ^]^

## 3 Integration of Wide Bandgap Semiconductors with 2D Materials

In this section, we introduce the integration techniques for forming WBG/2D heterostructures. Most 2D materials have a hexagonal in‐plane lattice structure similar to that of many WBGs. Some of their lattice constants are closely matched as shown in Figure 2h. More importantly, lattice‐matching requirements are not as stringent for integrating layered 2D materials, as will be discussed further in the following sections. Thus, the formation of heterostructures involving 2D materials and WBGs is feasible and promises various synergistic effects owing to their distinct advantages. For example, by combining the high structural and thermal stability of WBGs with the high carrier mobility and quantum confinement effects of 2D materials, the realization of high‐performance electronic and optical devices has become feasible. The different bandgaps and work functions of 2D materials and WBGs also enable the generation of energy barriers, thereby facilitating the control of electronic and structural properties to unveil new physical phenomena. Furthermore, WBG films grown on 2D materials are less affected by lattice mismatches during growth, resulting in a lower dislocation density. Additionally, the weak out‐of‐plane vdW interaction of 2D materials enables the facile transfer of the WBG film from the growth substrate to another substrate. Consequently, WBG/2D heterostructures are poised to drive significant innovations in the field of power conversion, optical components, electronic communications, and sensors,^[^ ^70^, ^71^ ^]^ motivating research endeavors toward these domains.

Techniques for fabricating WBG/2D heterostructures can be broadly categorized into two approaches: 1) forming 2D materials on WBGs and 2) forming WBGs on 2D materials (Figure 1). The fabrication technique for 2D materials on WBG was first reported in 2004, wherein epitaxial graphene (EG) was produced by the sublimation of silicon from SiC substrates at high temperatures.^[^ ^72^ ^]^ Since then, various fabrication techniques for WBG/2D heterostructures including dry and wet transfer techniques, pulsed laser deposition (PLD), and the annealing of transition metal thin films in a chalcogen atmosphere, have been employed.^[^ ^26^, ^73^, ^74^, ^75^ ^]^ Initially, the lattice orientation between the two materials was neither considered nor controlled. Later, to achieve well‐ordered WBG/2D heterostructures, epitaxial growth using chemical vapor deposition (CVD) has been reported.^[^ ^76^ ^]^ Additionally, solution‐based processes, such as solvothermal/hydrothermal synthesis, and liquid‐phase exfoliation, have been widely reported for the fabrication of 2D materials on WBGs.^[^ ^77^, ^78^, ^79^, ^80^, ^81^, ^82^, ^83^, ^84^ ^]^ However, these techniques are primarily employed to synthesize nanostructures, including nanosheets and nanoflowers, to maximize the exposed surface area. As a result, applications of these methods have been focused on developing sensors and catalysts.^[^ ^78^, ^79^, ^80^, ^81^, ^82^, ^83^, ^84^ ^]^

Techniques for fabricating WBGs on 2D materials have involved the development of several novel and previously unexplored concepts. In 2010, the growth of ZnO nanowalls and GaN films on defective graphene, referred to as “pinhole epitaxy”, was reported.^[^ ^28^ ^]^ Subsequently, quasi‐vdW (qvdW) epitaxy has been applied to form WBGs on 2D materials, as demonstrated by the growth of GaN on EG/SiC substrates.^[^ ^85^ ^]^ Unlike conventional epitaxy techniques, which typically involve strong ionic or covalent bonds between the epilayer and substrate, qvdW epitaxy is characterized by weak vdW interactions between vdW materials and thin films. The initial qvdW epitaxy technique yielded polycrystalline WBG thin films on 2D layers; however, recent advancements have led to the successful growth of single‐crystal WBG thin films by qvdW epitaxy. In 2017, a remote epitaxy technique was newly developed, wherein semi‐transparency of 2D materials allows the epitaxy to occur “remotely” through the 2D layer.^[^ ^86^ ^]^ Therefore, when thin films are remote epitaxially grown on crystalline substrates coated with 2D materials, the crystal orientation of the thin films follows that of the underlying substrate, not 2D materials. Remote epitaxy was first demonstrated by growing GaAs on a graphene‐coated GaAs substrate; this principle was subsequently applied to WBGs, such as GaN.^[^ ^87^ ^]^ By combining two growth techniques—2D material growth on WBGs and remote epitaxy on 2D materials—the successful growth of multiple stacks of WBG/2D structures was achieved in 2023, with all WBG layers crystallographically aligned.^[^ ^88^ ^]^ Below we discuss the various techniques developed for fabricating WBG/2D heterostructures and their distinct advantages, challenges, and prospects in detail.

### 3.1 2D Materials Formation on Wide Bandgap Semiconductors

#### 3.1.1 Transfer of 2D Materials

Owing to the weak interlayer vdW bonding in 2D materials, 2D layers can be easily isolated from the host substrate and transferred onto other substrates. This technique can be further classified into dry and wet transfer techniques depending on whether 2D materials are exposed to water or chemicals during the transfer process.^[^ ^88^ ^]^

**Figure 3:**
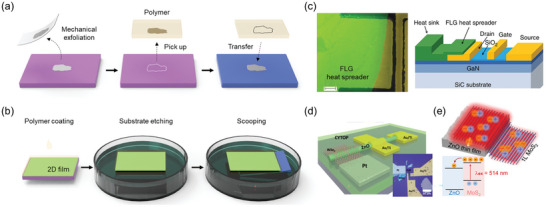
Transfer of 2D materials onto wide bandgap semiconductors. a,b) Schematics of (a) dry transfer and (b) wet transfer techniques. c) Optical and schematic images of few‐layer graphene transferred onto AlGaN/GaN heterostructure field‐effect transistors. Reproduced with permission.^[^ ^26^ ^]^ Copyright 2012, Nature Publishing Group. d) Schematic and optical images of 1D ZnO/2D WSe_2_ heterostructure‐based photosensors. Reproduced with permission.^[^ ^73^ ^]^ Copyright 2017, Wiley‐VCH. e) Schematic and band diagram of MoS_2_/ZnO heterostructure. Reproduced with permission.^[^ ^95^ ^]^ Copyright 2016, American Chemical Society.

The dry transfer technique is commonly employed for mechanically exfoliated 2D layers. As a support layer, polymer is affixed to a mechanically exfoliated 2D layer on the substrate. Utilizing the glass‐transition temperature of the polymer, the 2D layer is separated from the substrate at elevated temperatures and transferred onto another substrate (or material), and the polymer is either physically peeled off or chemically etched (**Figure** **3**a).^[^ ^89^ ^]^ This technique has been instrumental in fabricating various heterostructures based on 2D materials^[^ ^90^, ^91^, ^92^ ^]^ owing to the usage of mechanically exfoliated 2D layers, which avoid contamination by water or chemicals and result in high‐quality, clean interfaces in 2D‐based heterostructures. However, a glove box or vacuum chamber is often required to prevent the formation of air bubbles at the interface during the transfer process. Additionally, controlling the size and the thickness of the 2D layer during mechanical exfoliation poses challenges, limiting the size of 2D/WBG heterostructures.

In contrast, the wet transfer technique is primarily utilized for large‐area 2D layers. Similar to the dry transfer technique, polymer is coated onto the grown 2D layer as a support layer. The substrate is then delaminated in an etchant solution. Subsequently, the layer is rinsed several times with deionized water and scooped with the target substrate (Figure 3b).^[^ ^93^ ^]^ While this technique facilitates easy control of the size and thickness of 2D materials, it directly exposes the 2D layer to various chemicals and deionized water, increasing the likelihood of impurities forming at the heterostructure interface and risking damaging the 2D layer during the transfer process.^[^ ^94^ ^]^

Numerous studies on 2D/WBG heterostructures have been conducted by utilizing such transfer techniques. In 2012, Yan et al. employed wet transfer to incorporate few‐layer graphene into high‐power AlGaN/GaN heterostructure field‐effect transistors, in which graphene served as a heat spreader to address self‐heating issues (Figure 3c).^[^ ^26^ ^]^ Lee et al. utilized a dry transfer technique to fabricate mixed‐dimensional 1D‐ZnO/2D‐WSe_2_ heterojunctions and reported their potential applications as high‐performance photosensors (Figure 3d).^[^ ^73^ ^]^ Additionally, Kim et al. reported enhanced photoluminescence resulting from charge transfer at the interface of TMD/ZnO heterostructures by a wet transfer of TMDs onto ZnO thin films (Figure 3e).^[^ ^95^ ^]^ Although transfer techniques have been actively employed to study physical properties and various applications of 2D/WBG heterostructures, challenges such as size limitations and interface impurities persist. Therefore, developing transfer‐free processes is imperative for realizing scalable and reliable 2D/WBG heterostructures.

#### 3.1.2 Nonepitaxial Growth of 2D Materials on Wide Bandgap Semiconductors

**Figure 4:**
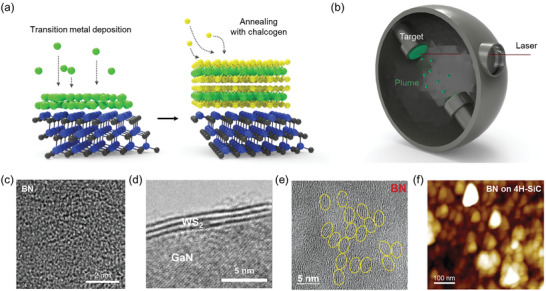
Nonepitaxial growth of 2D materials on wide bandgap semiconductors. a,b) Schematics showing (a) direct growth of TMDs on WBG through deposition of transition metal thin film followed by thermal annealing under chalcogen atmosphere and b) pulsed laser deposition (PLD) of 2D materials on WBG. c) High‐resolution transmission electron microscopy (HR‐TEM) image of an amorphous boron nitride layer grown on GaN substrate by molecular beam epitaxy. Reproduced with permission.^[^ ^30^ ^]^ Copyright 2023, Nature Publishing Group. d) Cross‐sectional HR‐TEM image of uniform tri‐layer WS_2_ film directly grown on GaN substrate. Reproduced with permission.^[^ ^74^ ^]^ Copyright 2022, Elsevier. e) Plan‐view HR‐TEM and f) AFM images of boron nitride grown on 4H‐SiC via PLD method. Yellow circles indicate the grain size of the boron nitride. Reproduced with permission.^[^ ^97^ ^]^ Copyright 2023, Wiley‐VCH.

To fabricate 2D/WBG heterostructures with clean interfaces, direct growth of 2D films on WBGs has been investigated. One reported method involves depositing a transition metal thin film on WBGs and annealing it in a chalcogen atmosphere to form TMD/WBG heterostructures (**Figure** **4**a).^[^ ^96^ ^]^ Another technique involves direct deposition of 2D materials using PLD. In PLD, a high‐energy laser is directed at the target to create plasma (also referred to as a plume). The plume particles are then adsorbed and diffused onto the surface of the WBG semiconductors, leading to the growth of 2D materials on the WBGs (Figure 4b).^[^ ^74^, ^97^ ^]^ Additionally, Kim et al. reported that boron nitride, which typically grows at temperatures above 1000 °C, can be directly grown on GaN at a relatively lower temperature of 680 °C using molecular beam epitaxy technique.^[^ ^30^ ^]^ Notably, the boron nitride grown on GaN exhibits an amorphous structure owing to the low growth temperature (Figure 4c). These direct growth techniques enabled the fabrication of large‐area 2D/WBG heterostructures with clean interfaces, and the deposition process could uniformly control the thickness of the 2D film on the WBG (Figure 4d).^[^ ^74^ ^]^ However, the grain size of the grown 2D materials typically ranged from a few nanometers to a few hundred nanometers (Figure 4c,e).^[^ ^30^, ^97^ ^]^ Therefore, the surface of the 2D/WBG heterostructure is often rough (Figure 4f), and the crystallinity is low, making it difficult to manifest the intrinsic properties of 2D materials.^[^ ^96^ ^]^

#### 3.1.3 Epitaxial Growth of 2D Materials on Wide Bandgap Semiconductors

**Figure 5:**
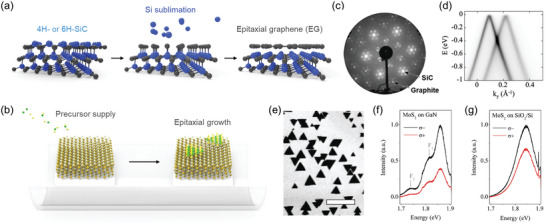
Epitaxial growth of 2D materials on wide bandgap semiconductors. a) Schematic illustration of EG formation on 6H‐SiC substrate via sublimation of Si at high temperature. b) Schematic illustration showing epitaxial growth of TMDs on WBGs via chemical vapor deposition (CVD). c) Low‐energy electron diffraction pattern and d) angle‐resolved photoelectron spectroscopy of EG on SiC substrate imply the epitaxial growth of graphene on SiC. Reproduced with permission.^[^ ^72^ ^]^ Copyright 2004, American Chemical Society. Reproduced with permission.^[^ ^102^ ^]^ Copyright 2011, National Academy of Sciences. e) Scanning electron microscopy (SEM) image of bidirectional triangular MoS_2_ grains grown on GaN showing the epitaxial relationship between MoS_2_ and GaN substrate. f,g) Circular polarized photoluminescence spectra of MoS_2_ on f) GaN and g) SiO_2_/Si substrates. Reproduced with permission.^[^ ^99^ ^]^ Copyright 2018, Wiley‐VCH.

Epitaxial growth techniques have also been utilized to fabricate high‐quality 2D/WBG heterostructures. In 1975, Van Bommel et al. discovered that when SiC is annealed at a high temperature (>800 °C) under ultra‐high vacuum (UHV) conditions, silicon sublimates, resulting in the formation of ultrathin EG on the SiC surface (**Figure** **5**a),^[^ ^98^ ^]^ making it the first method to fabricate epitaxial 2D/WBG heterostructures with high crystallinity and clean interfaces. However, unlike conventional epitaxy, wherein materials are grown bottom‐up by introducing precursors into a chamber, it is challenging to control the sublimation of Si atoms from the SiC substrate to leave behind carbon atoms that form uniform graphene layers. Therefore, challenges remain with this technique, such as the requirement for a UHV chamber and high‐temperature processes, and the difficulty in controlling the thickness of EG. Moreover, this technique is limited to fabricating EG/SiC heterostructure and is not applicable to other WBGs.

CVD is the most widely used technique for epitaxial growth of 2D materials on WBGs. In this approach, gas‐phase precursors are supplied to the hexagonal planes of WBGs with a small lattice mismatch with 2D materials, enabling the growth of 2D materials in accordance with the surface orientation of the WBGs (Figure 5b). This method allows the fabrication of large‐area, high‐quality 2D/WBG heterostructures. Since Ruzmetov et al. first epitaxially grew bidirectional MoS_2_ grains on GaN substrate, the epitaxial growth of various 2D/WBG heterostructures, including MoSe_2_/GaN, WS_2_/EG/SiC, and MoS_2_/EG/SiC, has been demonstrated.^[^ ^76^, ^99^, ^100^, ^101^ ^]^ However, several challenges need to be addressed. The WBGs must not chemically react with precursors under growth conditions, and their stability at high temperatures must be ensured.

As mentioned above, epitaxial growth techniques offer the advantage of fabricating large‐area, high‐quality 2D/WBG heterostructures. The alignment of the lattice orientation between SiC and the epitaxially grown graphite in the same direction on the millimeter scale was confirmed by low‐energy electron diffraction (LEED) measurements (Figure 5c).^[^ ^72^ ^]^ Additionally, a cone‐shaped band structure formed near the Fermi level was observed in angle‐resolved photoelectron spectroscopy (ARPES) measurements of EG (Figure 5d).^[^ ^102^ ^]^ These observations demonstrate that EG grown via Si sublimation has high crystal quality. Epitaxial growth using CVD techniques has predominantly been employed for the growth of TMDs on WBG. Triangular MoS_2_ grains aligned in opposite directions were grown on GaN substrate (Figure 5e), indicating that MoS_2_ grew following the lattice orientation of GaN. From the epitaxially grown MoS_2_/GaN heterostructures, an enhanced valley helicity of 0.33 at room temperature was observed (Figures 5f and g).^[^ ^99^ ^]^ Therefore, epitaxial growth of 2D layers on WBG is a promising method for fabricating large‐area, high‐quality 2D/WBG heterostructures.

### 3.2 Wide Bandgap Semiconductors Formation on 2D Materials

#### 3.2.1 Pinhole Epitaxy of Wide Bandgap Semiconductors

**Figure 6:**
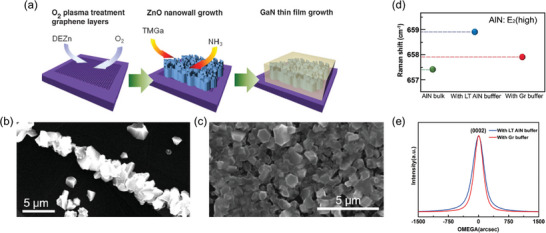
Pinhole epitaxy of wide bandgap semiconductors on 2D materials. a) Schematic of growth of ZnO nanowall and GaN thin films on O_2_‐plasma‐treated graphene. b,c) SEM images of GaN islands and film grown on graphene (b) without and (c) with O_2_ plasma treatment. Sparse GaN islands are grown on untreated graphene, whereas GaN film is successfully formed on plasma‐treated graphene. This implies that graphene defects formed by plasma treatment act as nucleation sites. Reproduced with permission.^[^ ^28^ ^]^ Copyright 2010, American Association for the Advancement of Science. d) Peak position of E_2_ phonon mode for (green) AlN bulk, (red) with a graphene buffer layer, and (blue) with low‐temperature AlN buffer layer showing strain relaxation of AlN on the graphene buffer layer. e) X‐ray rocking curves of AlN film grown on graphene buffer layer and low‐temperature AlN buffer layer. Reproduced with permission.^[^ ^103^ ^]^ Copyright 2020, Wiley‐VCH.

The growth of thin films on 2D materials is not straightforward, because the surface free energy of layered materials is much lower than conventional substrates. Therefore, the techniques for growing WBGs on 2D materials often involve novel approaches not previously known. In 2010, Chung et al. demonstrated the growth of ZnO nanowalls and GaN thin films on an O_2_ plasma‐treated graphene layer (**Figure** **6**a).^[^ ^28^ ^]^ They discovered that nucleation was suppressed when GaN was grown on untreated graphene (Figure 6b), whereas polycrystalline GaN films could be grown on the graphene layer by O_2_ plasma treatment (Figure 6c), indicating that GaN nucleation begins at the defect sites of the graphene layer formed by O_2_ plasma treatment. This technique has since evolved, leading to the development of pinhole epitaxy, where defect sites in the 2D layer on a single‐crystal substrate are utilized to achieve higher‐quality growth. Chang et al. grew AlN films on an N_2_ plasma‐treated graphene layer. The AlN film grown on the graphene showed the E_2_ phonon mode at 657.9 cm^−1^, which is very close to the strain‐free AlN peak position (657.4 cm^−1^). In contrast, the AlN film grown on the AlN buffer layer exhibited an E_2_ peak at 658.9 cm^−1^, indicating a high compressive strain (Figure 6d).^[^ ^103^ ^]^ The narrow X‐ray rocking curve of AlN further proves the higher crystal quality of the AlN film on the graphene layer (Figure 6e), demonstrating that the interfacial graphene layer effectively alleviated the strain to achieve a high‐quality AlN film. To investigate the underlying mechanism, Journut et al. observed the interface between GaN islands and a graphene/SiC substrate. Cross‐sectional TEM observations revealed that the nucleation site of the GaN island was directly connected to the SiC substrate through the defect regions of graphene, providing direct evidence of pinhole epitaxy.^[^ ^104^ ^]^ Pinhole epitaxy leverages precise control of defect sites to guide the growth of WBG, thereby improving the alignment and crystallinity of the resulting heterostructures.

#### 3.2.2 Quasi‐van der Waals Epitaxy on 2D Materials

**Figure 7:**
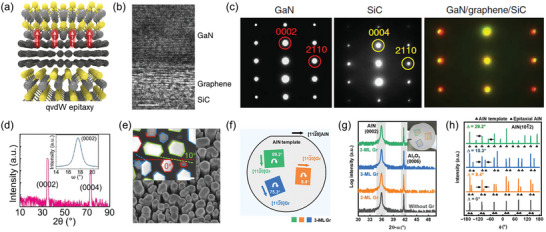
Quasi‐vdW (qvdW) epitaxy of wide‐bandgap semiconductors on 2D materials. a) Schematic of qvdW epitaxy on 2D materials. b) Cross‐sectional HR‐TEM image of GaN grown on EG/SiC substrate. c) Selected area electron diffraction patterns obtained from GaN, SiC, and the interface of GaN/graphene/SiC, indicating the epitaxial relationship of GaN, SiC, and graphene. Reproduced with permission.^[^ ^85^ ^]^ Copyright 2014, Nature Publishing Group. d) X‐ray diffraction (XRD) pattern and e) SEM image of GaN nanorods grown on graphene/amorphous glass substrate. The inset shows the X‐ray rocking curve of (0002) for GaN and the high‐resolution SEM image of the nanorods. The preferred orientations of 0°, 10°, and 30° are indicated by red, green, and blue lines, respectively. Reproduced with permission.^[^ ^106^ ^]^ Copyright 2021, American Association for the Advancement of Science. f) Schematic of transferred 3L graphene films on AlN substrate. The graphene s have different rotational angles of 8.4° (orange), 15.3° (blue), and 29.2° (green), respectively. g) XRD 2θ‐ω scans and h) ϕ scans of AlN film grown on each graphene film. Different in‐plane orientations are observed in XRD ϕ scans, whereas 2θ‐ω scans show identical out‐of‐plane orientations. This implies that the orientations of the AlN films are strongly affected by the graphene orientation. Reproduced with permission.^[^ ^107^ ^]^ Copyright 2023, American Association for the Advancement of Science.

In quasi‐van der Waals epitaxy, the crystal orientation of the as‐grown thin films is epitaxially aligned with that of the 2D layer, unlike that in the pinhole epitaxy (**Figure** **7**a). In 1985, Koma et al. first reported the concept of vdW epitaxy by growing a Se layer on a Te layer to form a 2D/2D heterostructure.^[^ ^105^ ^]^ Since then, several materials, particularly 2D materials, have been grown using vdW epitaxy. In 2012, Kim et al. demonstrated the fabrication of WBG/2D heterostructure by growing GaN on an EG/SiC substrate, utilizing quasi‐vdW epitaxy (Figure 7b), where the term “quasi” is employed to denote that the grown materials are thin films rather than layered materials.^[^ ^85^ ^]^ However, determining the origin of the GaN lattice orientation was difficult because the orientations of the EG, SiC, and grown GaN layers were identical (Figure 7c). Later studies found that both qvdW epitaxy and remote epitaxy of GaN can occur on EG/SiC substrates, with the epitaxy mode largely dependent on growth conditions and the thickness of the EG. Determining the epitaxy mode is more straightforward if the substrate and graphene layer do not exhibit crystallographic relationships. For example, a recent study by Ren et al. showed that nitride films grown on a graphene/amorphous glass substrate exhibit specific in‐plane lattice orientations (0°, 10°, and 30°), confirming that the epilayer follows the orientation of the graphene layer because of qvdW epitaxy mechanisms (Figure 7d,e).^[^ ^106^ ^]^ Furthermore, Liu et al. transferred tri‐layer (3L) graphene onto a 2‐inch AlN (0001) wafer with different rotational angles (8.4°, 75.3°, and 89.2°) and subsequently grew an AlN film to investigate the epitaxial relationship (Figure 7f).^[^ ^107^ ^]^ The resulting single‐crystal AlN films on each graphene layer retained the same (0001) out‐of‐plane orientation as the AlN wafer but exhibited in‐plane orientations of 8.4°, 15.3°, and 29.2° (Figure 7g,h). This alignment corresponds closely with the rotational angles of the transferred graphene, demonstrating that the AlN film grew in the qvdW epitaxy mode on the 3L graphene. While qvdW epitaxy can allow for a large‐scale growth of high‐quality WBG/2D heterostructures, the crystal quality of the WBGs is highly dependent on the quality and thickness of the underlying 2D layer. This requirement poses a challenge because high‐quality, large‐area 2D substrates are necessary.

#### 3.2.3 Remote Epitaxy via 2D Materials

**Figure 8:**
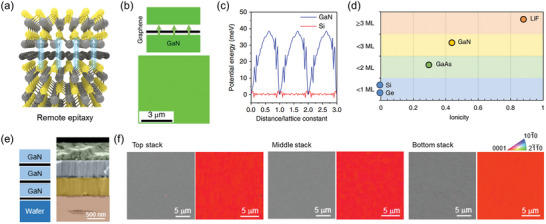
Remote epitaxy of wide bandgap semiconductors through 2D materials. a) Schematic of remote epitaxy through atomically thin 2D materials. b) Upper panel: Schematic image of the remote epitaxy of GaN on monolayer graphene/GaN substrate. Bottom panel: electron back‐scattered diffraction (EBSD) map image of GaN film on monolayer graphene/GaN substrate formed via remote epitaxy. c) Potential fluctuation profiles obtained from (blue) graphene/GaN and (red) graphene/Si substrates. d) Schematic of the remote interaction depth depending on the ionicity of various substrates, indicating that the remote epitaxy is affected by the ionicity of the substrate. Reproduced with permission.^[^ ^108^ ^]^ Copyright 2018, Nature Publishing Group. e) Schematic and corresponding cross‐sectional SEM image of GaN/BN multiple stack structure. f) Plan‐view SEM images and EBSD mapping images obtained from the top, middle, and bottom stack. The identical color contrast in EBSD mapping images implies the same orientation of each stack in multiple stack structures. Reproduced with permission.^[^ ^30^ ^]^ Copyright 2023, Nature Publishing Group.

Unlike qvdW epitaxy, remote epitaxy involves the growth of an epilayer following the orientation of the substrate rather than the 2D layer (**Figure** **8**a). In 2017, Kim et al. reported the concept of remote epitaxy by growing a single‐crystal GaAs epilayer on a GaAs substrate with a monolayer of transferred graphene.^[^ ^86^ ^]^ Subsequent research elucidated the mechanism, demonstrating that the fluctuating potential ofpolar substrates, for example, GaN, can penetrate an atomic‐thick 2D layer (particularly graphene), enabling remote epitaxy through the 2D layer (Figure 8b,c).^[^ ^108^ ^]^ They investigated the epitaxial growth behavior by transferring 1–3L of graphene onto Si, Ge, GaAs, GaN, and LiF substrates with different ionicities. The results showed that polycrystalline films grew even with a single graphene layer on low ionicity substrates such as Si and Ge. In contrast, on the high ionicity LiF substrate, the potential fluctuation of the substrate could penetrate through up to 3L of graphene, enabling the growth of single‐crystal films via remote epitaxy (Figure 8d).^[^ ^108^ ^]^

More recent reports have suggested that remote epitaxy may also be achieved using amorphous 2D materials, such as amorphous carbon and boron nitride, not just a crystalline 2D layer, thus significantly increasing its universality.^[^ ^30^ ^]^ Considering that amorphous 2D materials can be grown at the wafer scale (i.e., free of transfer processes) on WBGs, the utilization of amorphous 2D materials makes remote epitaxy a promising technique for growing scalable single‐crystalline WBGs. In addition, the benefits of the transfer‐free fabrication technique were exploited to grow multiple stacks of 3D/2D heterostructures (Figure 8e). They confirmed that each GaN layer exhibited an identical orientation (Figure 8f), which verifies the successful fabrication of a GaN/BN multiple stack structure by remote epitaxy.^[^ ^30^ ^]^

Challenges in conducting remote epitaxy arise from the attenuation of the surface potential of the substrate by the 2D layers. 2D materials must be thin enough (typically 1–2L) to allow remote epitaxy phenomena. Preserving clean interfaces is also critical because interfacial contamination can screen the surface potential. Therefore, stringent process control is necessary for the growth of high‐quality thin films on 2D materials via remote epitaxy.

#### 3.2.4 Advantages of Epitaxy on 2D Materials

**Figure 9:**
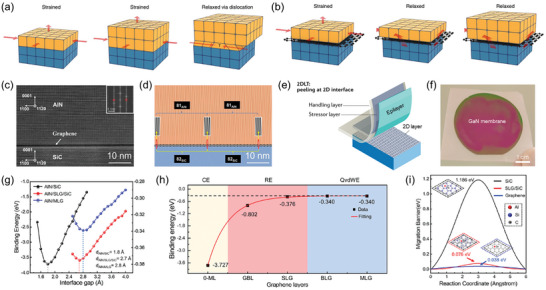
Advantages of epitaxial growth on 2D materials. a,b) Schematics showing strain relaxation by (a) dislocation formation in conventional epitaxy and (b) spontaneous relaxation on 2D materials. Reproduced with permission.^[^ ^25^ ^]^ Copyright 2020, Nature Publishing Group. c) Cross‐sectional high‐angle annular dark field scanning electron microscopy image and d) corresponding inverse fast Fourier transform image of the AlN/graphene/SiC heterostructure. 81 AlN stripe spacings matched with 82 SiC stripe spacings, implying that the strain of AlN is well relaxed by the interfacial graphene layer. Reproduced with permission.^[^ ^109^ ^]^ Copyright 2023, American Chemical Society. e) Schematic image of 2D material‐based layer transfer (2DLT) technique. Reproduced with permission.^[^ ^110^ ^]^ Copyright 2022, Nature Publishing Group. f) Photograph of a 2‐inch GaN membrane exfoliated from BN/GaN substrate via 2DLT. Reproduced with permission.^[^ ^30^ ^]^ Copyright 2023, Nature Publishing Group. g) Interfacial binding energies of AlN/SiC (black line), AlN/single‐layer graphene(SLG)/SiC (red line), and AlN/multilayer graphene(MLG) (blue line), as a function of the heterointerface gap. h) Comparison of binding energy of SiC substrates covered with different layers of graphene. i) Calculated migration barrier of Al atom on SiC (black), SLG/SiC (red), and graphene (blue). Reproduced with permission.^[^ ^109^ ^]^ Copyright 2023, American Chemical Society.

Notably, epitaxy on 2D layers enables new degrees of freedom in materials engineering and heterogeneous integration. First, the epitaxy of 3D materials on 2D layers addresses strain issues inherent in conventional heteroepitaxy. Conventional epitaxy involves strong interactions (such as covalent or ionic bonds) between the substrate and epilayer, often leading to strain and dislocations owing to lattice mismatch (**Figure** **9**a).^[^ ^25^ ^]^ In contrast, epilayers grown on dangling‐bond‐free 2D layers exhibit vdW interactions, alleviating the strain (Figure 9b). Recently, Wang et al. demonstrated the growth of AlN on a monolayer graphene/SiC substrate via remote epitaxy with interfacial strain relaxation. Cross‐sectional high‐angle annular dark‐field scanning electron microscopy (HAADF‐STEM) images and the corresponding inverse fast Fourier transform (FFT) lattice fringes revealed that 81 AlN stripe spacings matched well with 82 SiC stripe spacings (Figure 9c,d),^[^ ^109^ ^]^ directly demonstrating that the presence of graphene at the interface effectively relieves the strain in the epilayer. Second, such weak vdW interactions at the interface enable the exfoliation and transfer of the epilayer to other substrates—called a 2D material‐based layer transfer (2DLT) technique (Figure 9e).^[^ ^110^ ^]^ The simple mechanical exfoliation of single‐crystalline WBG membranes was demonstrated using the 2DLT technique (Figure 9f).^[^ ^30^ ^]^ The strain relaxation in WBGs grown on 2D layers and the benefits of 2DLT can be attributed to the significantly lower interfacial binding energy in remote and qvdW epitaxy, as calculated by Wang et al. using density functional theory calculations, compared to conventional epitaxy (Figure 9g,h).^[^ ^109^ ^]^ Additionally, the reduced migration barrier on 2D layers enlarges WBG grain size, leading to the formation of fewer dislocations and facilitating the growth of high‐quality WBG films (Figure 9i). The improved crystal quality of WBG films grown on 2D layers has been evaluated via XRD rocking curves.^[^ ^103^, ^104^, ^106^ ^]^ Moreover, recent studies employing plan‐view SEM, TEM imaging, and cathodoluminescence have revealed that the dislocation density in WBG films grown on 2D layers is significantly reduced by a factor of two to an order of magnitude‐compared to those grown by conventional epitaxy on 3D substrates.^[^ ^25^, ^103^, ^109^, ^111^ ^]^ It is important to note that the quality of as‐grown WBG films is strongly influenced by various factors, such as the quality of 2D layers, interfaces, and nucleation conditions of WBG films. It is thus necessary to develop a holistic approach, combining theoretical studies, ideal 2D materials templates, and nucleation conditions, to quantitatively study the impact of 2D materials on the dislocation formation mechanisms. Furthermore, recycling of substrates for subsequent growth is also possible because exfoliation occurs precisely at 2D material interfaces without any substrate damage. Therefore, 2DLT of WBG membranes represents a cost‐effective and sustainable approach for diverse applications.

**Figure 10:**
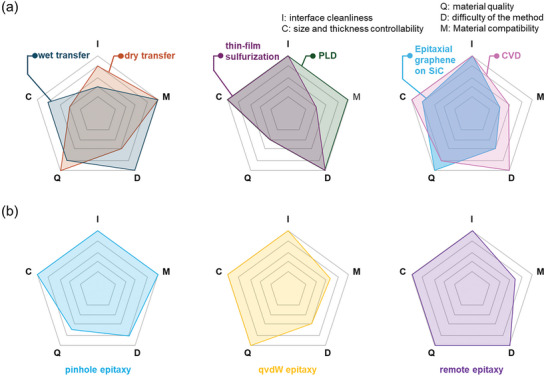
Summary of strengths and weaknesses of fabrication techniques for WBG/2D heterostructures.

In this section, we described various techniques for fabricating WBG/2D heterostructures. We also discussed advantages and disadvantages of each technique, which are summarized in **Figure** **10** and **Table** **1**. Although easily accessible transfer techniques and nonepitaxial growth methods have been widely reported, issues with interfacial contamination, scalability, and quality remain in these transfer‐based approaches and nonepitaxial approaches. In contrast, epitaxy techniques for directly growing 2D layers and WBGs offer a promising approach, enabling the fabrication of large‐area WBG/2D heterostructures with clean interfaces. Hence, advancing the growth processes is expected to enable the possibility of growing high‐quality WBG/2D heterostructures as well as various multiple quantum wells (MQWs) and superlattices utilizing various 2D layers and WBGs.

**Table 1:** Comparison of fabrication techniques for WBG/2D heterostructures in terms of interface cleanliness, size and thickness controllability, material quality, difficulty of the method, and material compatibility.

Category	Technique	Interface cleanliness	Size and thickness controllability	Material quality	Difficulty of the method	Material compatibility
2D on WBG	Dry transfer	Bubbles, wrinkles	Difficult to control the size and thickness of 2D layers	Very good	Difficult to obtain specific 2D layers	Very good
	Wet transfer	Bubbles, wrinkles, and chemical contaminants	Very good	Good	Not difficult	Very good
	PVD	Clean	Very good	Poor	Not difficult	Very good
	Sulfurization of transition metal thin film	Clean	Very good	Poor	Not difficult	Limited to TMDs
	Epitaxial graphene on SiC	Clean	Difficult to control the thickness	Very good	High temperature & UHV conditions are required.	Limited to SiC and graphene
	CVD	Clean	Very good	Good	Not difficult	Difficult to grow graphene and hBN
WBG on 2D	Pinhole epitaxy	Clean	Very good	2D layer is not pristine	Precise control of defects in a 2D layer is required	Very good
	qvdW epitaxy	Clean	Very good	Very good	High‐quality 2D layers are required	Limited to materials with hexagonal structure
	Remote epitaxy	Clean	Very good	Very good	Not difficult	Limited by ionicity and screening

## 4 Applications of WBG/2D Heterostructures

Applications of WBG/2D heterostructures can be broadly categorized into three main types. The first category includes applications that exploit the electronic structures of the WBG/2D heterojunctions. The second category involves the utilization of 2D materials to improve the performance of WBG devices. The third category encompasses utilizing 2DLT to detach WBGs from their original substrates and integrate them into other platforms to enable new device applications.

### 4.1 WBG/2D Heterojunction Devices

**Figure 11:**
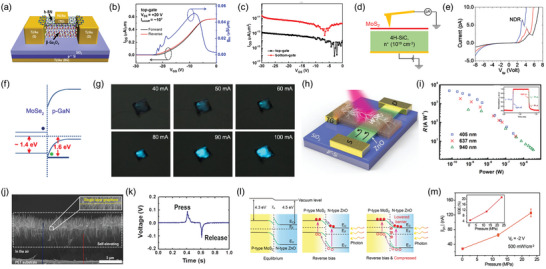
WBG/2D heterojunction‐based applications. a) Schematic of metal‐insulator‐semiconductor field‐effect transistor (MISFET) based on hBN/β‐Ga_2_O_3_ heterostructure. b) I_DS_‐*g_m_*‐V_GS_ transfer characteristics of the hBN/β‐Ga_2_O_3_ heterostructure MISFET, with the hBN layer employed as a top‐gate dielectric layer. c) Gate leakage currents under top‐gate (hBN) and bottom‐gate (SiO_2_) operation show a high breakdown voltage using hBN dielectric. Reproduced with permission.^[^ ^112^ ^]^ Copyright 2017, American Chemical Society. d) Schematic of conductive AFM setup to probe current transport through MoS_2_/n^+^ 4H‐SiC heterojunction. e) *I–V* characteristic of MoS_2_/n^+^ 4H‐SiC heterojunction showing Esaki diode behavior. Reproduced with permission.^[^ ^75^ ^]^ Copyright 2022, Wiley‐VCH. f) Energy band diagram and of the MoSe_2_/GaN heterostructure. g) Photographs obtained from MoSe_2_/GaN light‐emitting diode (LED) under different injection currents. Reproduced with permission.^[^ ^115^ ^]^ Copyright 2016, American Chemical Society. h) Structure and operating mechanism of WSe_2_/ZnO light‐driven junction field‐effect transistor (LJFET). i) Responsivities of the LJFET as a function of light power under 405, 637, and 940 nm illumination. The inset displays a temporal response of ≈10 µs from the device under 637 nm illumination. Reproduced with permission.^[^ ^125^ ^]^ Copyright 2020, Wiley‐VCH. j) SEM image of an epitaxial ZnO/graphene/ZnO double heterostructure. k) Piezoelectric output voltage of the device under compress‐release conditions. Reproduced with permission.^[^ ^126^ ^]^ Copyright 2015, Elsevier. l) Band diagrams of the MoS_2_/ZnO p‐n junction diode under equilibrium, reverse bias, and reverse bias with compressive pressure. m) Photocurrent of the p‐n junction diode as a function of applied pressure. The inset shows the external quantum efficiency of the device under different pressures. Reproduced with permission.^[^ ^127^ ^]^ Copyright 2016, Wiley‐VCH.

Applications based on WBG/2D heterojunctions have been reported for a variety of electronic and optoelectronic devices, as well as novel devices. In the field of electronic applications, heterojunction devices leverage the properties of the constituent materials or the emergent properties of heterointerfaces. In 2017, Kim et al. fabricated a qvdW heterostructure by combining β‐Ga_2_O_3_ semiconducting channel, which can be cleaved in the (100) direction, with the 2D insulator hBN (**Figure** **11**a).^[^ ^112^ ^]^ They demonstrated the application of this heterostructure in a metal‐insulator‐semiconductor field‐effect transistor (MISFET). They investigated the improvement of FET performance by using hBN as a top‐gate dielectric when compared with SiO_2_ as a bottom‐gate dielectric in devices with the same channel layer (β‐Ga_2_O_3_). The results showed that top‐gate FETs using hBN dielectric exhibited a higher threshold voltage and an on/off ratio of ≈10^7^ compared to the case of using SiO_2_ dielectric (Figure 11b). In addition, hBN dielectric exhibited a lower leakage current (Figure 11c). Furthermore, they demonstrated that the subthreshold swing and threshold voltage can be controlled through dual‐side gating. Similarly, a quasi‐vdW heterostructure has been fabricated by transferring n‐type β‐Ga_2_O_3_ and p‐type semiconducting 2D WSe_2_, enabling the implementation of a junction field‐effect transistor (JFET).^[^ ^113^ ^]^ The p‐channel JFET exhibits a low subthreshold swing of 133 mV dec^−1^ and a high on/off ratio of ≈10^8^.

In addition to exploring the lateral carrier transport in WBG/2D heterostructures, electronic devices using the carrier transport in the vertical direction have been studied. In 2018, Yan et al. cleaved and transferred (100) oriented β‐Ga_2_O_3_, known for its high threshold voltage, onto graphene to fabricate a β‐Ga_2_O_3_/graphene vertical heterostructure.^[^ ^114^ ^]^ Using this heterostructure, they fabricated a vertical transistor with a high on/off ratio of 10^4^. Furthermore, the vertical transistor exhibited an exceptionally high breakdown electric field of 5.2 MV cm^−1^, surpassing other conventional materials such as Si, 6H‐SiC, diamond, and even lateral devices based on β‐Ga_2_O_3_. As another example of a vertical device, an Esaki diode was demonstrated using a MoS_2_/4H‐SiC heterostructure.^[^ ^75^ ^]^ An MoS_2_ layer was directly grown by depositing a Mo film on an n^+^ 4H‐SiC substrate and annealing it in a sulfur atmosphere. The vertical transport characteristics of the MoS_2_/4H‐SiC heterostructure were characterized using conductive atomic force microscopy (AFM), through a clean interface achieved via direct growth (Figure 11d). Conductive AFM measurements revealed a significant negative differential resistance (NDR) arising from band‐to‐band tunneling, confirming the behavior of the Esaki diode in the forward bias region (Figure 11e).

Among various applications, photodetectors and electroluminescence (EL) devices have been the most extensively studied because of the distinctive band properties of 2D materials and WBGs. In 2016, Chen et al. epitaxially grew MoSe_2_, an n‐type 2D semiconductor, on a GaN substrate, to fabricate high‐quality, large‐area MoSe_2_/GaN heterostructures.^[^ ^115^ ^]^ In the heterostructure, two EL peaks were clearly observed at 3.0 and 1.45 eV, corresponding to the bandgaps of GaN and MoSe_2_, respectively (Figure 11f). LEDs fabricated from the heterostructure yielded blue emission because of the weaker emission from the MoSe_2_ layer than from GaN. Although the power conversion efficiency was low (≈1.29%), the brightness varied with the injection current, demonstrating the potential applications of the WBG/2D heterostructures for emitters (Figure 11g).

Numerous studies have explored UV detectors by combining the high carrier mobility of graphene with the bandgap of WBGs.^[^ ^116^, ^117^, ^118^ ^]^ In 2016, Kong et al. fabricated a heterojunction device by transferring a large‐area graphene layer, grown via CVD, onto a Sn‐doped n‐type β‐Ga_2_O_3_ wafer.^[^ ^116^ ^]^ The device displayed rectifying characteristics under deep ultraviolet (DUV) light (254 nm) illumination, corresponding to the bandgap of β‐Ga_2_O_3_. This behavior originates from the built‐in electric field formed by band bending at the interface between graphene and β‐Ga_2_O_3_. The fabricated device exhibited a responsivity of 39.3 A W^−1^, a detectivity of 5.92 × 10^13^ cm Hz^1/2^ W^−1^, and a high external quantum efficiency of 1.96 × 10^4^ compared to other Ga_2_O_3_‐based DUV photodetectors.^[^ ^119^, ^120^, ^121^ ^]^ It also showed excellent stability and reproducibility under prolonged DUV light exposure, demonstrating the potential applications of the WBG/graphene heterostructure.

In addition to photodetectors that solely utilize the bandgap of WBGs, photodetectors capable of detecting wavelengths corresponding to the bandgaps of both WBGs and 2D materials have been explored.^[^ ^73^, ^122^, ^123^, ^124^ ^]^ These examples demonstrate that optoelectronic devices utilizing WBG/2D heterostructures have been actively studied to leverage the distinct bandgaps of each material. However, several challenges persist, including emission and detection imbalances due to the disparity in scales between the two materials, as well as trapping issues at their interfaces. There is a clear need for further exploration of the interlayer carrier‐transport mechanism in WBG/2D heterostructures. Understanding these mechanisms is pivotal for advancing the development and application of optoelectronic devices, particularly those that utilize MQWs or multiple heterostructures.

In addition to the devices based on well‐known principles, novel device concepts have also been reported. In 2019, Guo et al. proposed the fabrication of a light‐driven junction field‐effect transistor (LJFET) using an n‐type ZnO belt as a channel layer and a p‐type WSe_2_ nanosheet as a photoactive top‐gate material (Figure 11h).^[^ ^125^ ^]^ The LJFET operates based on the principle that photoexcited carriers in WSe_2_ under light illumination create an enlarged depletion region within the ZnO channel, resulting in a dramatic increase in channel resistance, which triggers the device's response. The LJFET exhibited high efficiency, with a responsivity of up to 1.3 × 10^3^ A W^−1^ and a fast response time of ≈10 µs, across the range from near‐infrared to UV wavelengths (Figure 11i). In 2015, Shin et al. utilized defects in a graphene layer to facilitate hydrothermal synthesis.^[^ ^126^ ^]^ They grew ZnO nanorods (NRs) on both sides of the graphene layer, thereby fabricating a ZnO NRs/graphene/ZnO NRs double heterostructure (Figure 11j). They transferred this double heterostructure onto a flexible ITO/PET substrate and proposed that the combined piezoelectric effect of the two ZnO NRs layers could be employed as a nanogenerator with enhanced performance (Figure 11k). Xue et al. demonstrated the piezophotonic effect by fabricating a photodiode using a heterojunction of n‐type ZnO and SF_6_ plasma‐treated p‐type MoS_2_.^[^ ^127^ ^]^ The resulting photodiode exhibited excellent rectifying behavior. Applying pressure affected the interfacial electronic structure, thereby altering the performance of the diode (Figure 11l). This led to a novel demonstration of the piezophotonic effect, in which the external quantum efficiency of the photodiode was enhanced four times under a pressure of ≈23 MPa (Figure 11m).

Thus, WBG/2D heterostructures have been applied in various fundamental electronic, optoelectronic, and energy devices, as well as novel device concepts for revolutionary performances. However, while various electronic device applications such as hot electron transistors and high‐frequency electronics in the terahertz range have been investigated,^[^ ^128^, ^129^ ^]^ exceptional results have not yet been achieved, likely because most devices have attempted to utilize carrier transport in the out‐of‐plane direction, focusing on vertical heterostructures, rather than exploiting the unique properties of 2D materials that manifest in the in‐plane direction. Therefore, developing new device structures that can exploit these unique properties is crucial for advancing this field. Additional challenges for advancing WBG/2D heterostructure‐based devices include the limitations of fabrication techniques and the lack of understanding of the physical phenomena at the interface such as charge transfer and electronic structure changes. Therefore, the development of advanced fabrication techniques in the future will open new avenues of high‐performance WBG/2D heterostructure applications.

### 4.2 Improving the Performance of WBG Devices by 2D Materials

**Figure 12:**
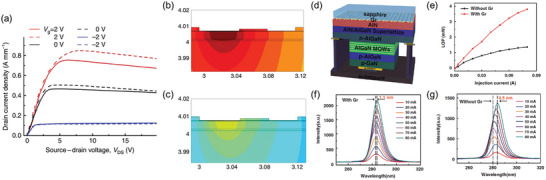
WBG devices with additional functionality by 2D materials. a) *I*_DS_–*V*_DS_ characteristics of AlGaN/GaN high‐power field‐effect transistors (HFETs) with (dashed lines) and without (solid lines) a graphene heat spreading layer. b,c) Temperature profiles in AlGaN/GaN HFETs on sapphire substrate powered at 3.3 W mm^−1^ (b) without and (c) with a heat spreader, implying that the heat spreader effectively reduces the operation temperature of HFETs. Reproduced with permission.^[^ ^26^ ^]^ Copyright 2012, Nature Publishing Group. d) Schematic image of a III‐N MQW LED grown on a graphene/sapphire substrate. e) Light output power (LOP) of the MQW LEDs, grown with/without graphene, as a function of injection currents. f,g) EL spectra of the LEDs, grown (f) with and (g) without graphene, under different injection currents. The small shift of EL spectra indicates that the III‐N MQW LED grown on the graphene layer possesses high crystallinity. Reproduced with permission.^[^ ^130^ ^]^ Copyright 2022, Nature Publishing Group.

Owing to the distinct advantages of 2D materials and WBGs, leveraging unique properties of 2D materials have also been widely studied to address the challenges in WBG‐based applications. In 2012, Yan et al. addressed the self‐heating issue of high‐power AlGaN/GaN heterostructure field‐effect transistors (HFET) by fabricating graphene/AlGaN/GaN structures.^[^ ^26^ ^]^ Graphene, which exhibits a high thermal conductivity, was transferred onto an AlGaN/GaN heterostructure and acted as an effective heat‐spreading layer. This approach resulted in a maximum drain current density increase of up to 12% (**Figure** **12**a). Simulations further demonstrated that the maximum temperature in an AlGaN/GaN HFET without a heat spreader was 181 °C (Figure 12b), whereas incorporating a graphene heat spreader reduced the temperature to ≈113 °C (Figure 12c). The significant reduction in temperature demonstrates that effective thermal management using 2D materials can substantially improve device performance.

Recently, it was revealed that high‐quality WBG films with low dislocation densities can be grown on 2D layers, and research utilizing this approach is actively progressing. For example, Chang et al. demonstrated that growing AlN on N_2_ plasma‐treated graphene/sapphire substrates resulted in lower density of dislocations.^[^ ^130^ ^]^ They applied this finding to fabricating DUV LEDs with high‐quality AlGaN MQWs (Figure 12d). The DUV LEDs grown on graphene exhibited more than twice the light output power compared to the LEDs directly grown on sapphire (Figure 12e). The LEDs also exhibited smaller shift and less broadening of EL signals (Figure 12f,g), effectively mitigating the performance degradation originating from dislocations. Additionally, other strategies have been attempted in various directions, including using 2D materials to form Ohmic contacts on WBGs.^[^ ^131^, ^132^ ^]^ However, most studies remain at the proof‐of‐concept level and often rely on transfer methods. Considering that the properties of 2D materials, WBGs, and their interfaces collectively affect device performance, developing and applying scalable and controllable processes to integrate 2D materials with WBGs are necessary.

### 4.3 WBG Applications Enabled by 2D Materials‐based Layer Transfer

**Figure 13:**
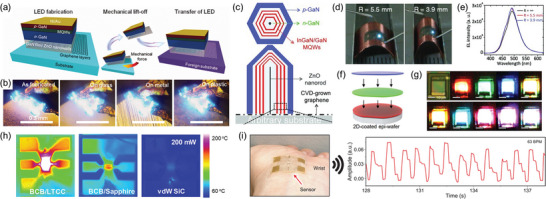
WBG applications via 2D materials‐based layer transfer (2DLT). a) Transfer process of multiple quantum well (MQW)/GaN film/ZnO nanowalls directly grown on a defective graphene layer. b) Photographs of MQW LED operation before and after transfer onto different substrates. Reproduced with permission.^[^ ^28^ ^]^ Copyright 2010, American Association for the Advancement of Science. c) Schematic structure of InGaN/GaN MQW/ZnO nanorods grown on defective graphene. d) Photographs and e) EL spectra of the flexible InGaN/GaN MQW/ZnO LEDs under different bending radii. Reproduced with permission.^[133^^]^ Copyright 2011, Wiley‐VCH. f) Schematic of vertical stacking of red, green, and blue layers via 2DLT. g) Electroluminescence microscopy images of vertically stacked micro‐LEDs illuminating red, green, blue, yellow, orange, cyan, pink, purple, and white light via mixing RGB colors. Reproduced with permission.^[^ ^137^ ^]^ Copyright 2023, Nature Publishing Group. h) Temperature maps of AlGaN/GaN high electron mobility transistors (HEMTs) (via infrared camera) at 200 mW on various substrates. Reproduced with permission.^[^ ^138^ ^]^ Copyright 2020, American Chemical Society. i) Photograph and wireless pulse signals obtained from a transferable GaN surface acoustic wave device. Reproduced with permission.^[^ ^29^ ^]^ Copyright 2022, American Association for the Advancement of Science.

Weak vdW interactions between 2D materials and thin films allow the facile exfoliation of thin films from 2D materials. Such a 2DLT technique provides a unique opportunity to produce ultrathin WBG membranes that can be integrated into arbitrary platforms for novel applications. In 2010, Chung et al. demonstrated a transferable LED by growing ZnO nanowalls and GaN thin films on top of an O_2_ plasma‐treated graphene layer and then performing a mechanical lift‐off of the layer (**Figure** **13**a,b).^[^ ^28^ ^]^ Later, Lee et al. fabricated ZnO nanorods and InGaN/GaN MQW nanorods on defective graphene using pinhole epitaxy (Figure 13c) and transferred them to a flexible substrate to achieve a flexible blue LED.^[^ ^133^ ^]^ The fabricated flexible LED demonstrated remarkable stability, maintaining the same EL intensity as in its flat state, even at a curvature radius of *R* = 3.9 mm (Figure 13d,e). In 2012, Kobayashi et al. successfully grew large‐area, centimeter‐scale III‐N MQW thin films on hBN.^[^ ^134^ ^]^ Employing the weak vdW interaction between the thin film and the hBN layer, the MQW thin film was transferred onto an indium sheet. The transferred MQW thin film demonstrated an exceptionally bright blue emission at room temperature, with an EL intensity more than twice that of conventional MQW LEDs. In addition, Karrakchou et al. successfully grew large‐scale hBN film on a sapphire substrate, followed by the growth of III‐N MQWs, to fabricate transferable LEDs.^[^ ^135^ ^]^ They later expanded this demonstration to a large area of 6 inches.^[^ ^136^ ^]^ Recently, vertical full‐color micro‐LEDs have been fabricated using the 2DLT technique.^[^ ^137^ ^]^ Shin et al. grew various III‐V and III‐N compound semiconductor‐based LED layers, that operate in red, green, and blue regimes on 2D materials. Using 2DLT, they isolated LED layers of each color from the substrates and then stacked the LED layers to form vertical full‐color micro‐LEDs (Figure 13f). The fabricated micro‐LEDs exhibited not only red, green, and blue emissions, but also the ability to control the injection current in each layer, enabling a spectrum of colors, including yellow, orange, cyan, pink, purple, and white, with extremely high pixel density (Figure 13g).^[^ ^137^ ^]^

In addition to LEDs, transferable WBGs via WBG/2D heterostructures have been employed in diverse applications such as high electron mobility transistors (HEMTs), sensors, and wearable electronics. Hiroki et al. demonstrated that growing AlGaN/GaN films on hBN and transferring them onto a copper plate via 2DLT could effectively mitigate self‐heating issues in HEMTs. HEMTs grown on a sapphire substrate typically exhibited a temperature rise of up to 50 °C (Δ*T* of 27 °C) in on‐state, whereas those transferred to a copper plate showed only a 30 °C rise (Δ*T* of 7 °C), demonstrating significantly enhanced thermal management capabilities.^[^ ^27^ ^]^ In addition, Motala et al. transferred AlGaN/GaN HEMTs to various substrates, including sapphire, SiC, and even adhesive tapes, using hBN as a release layer (Figure 13h).^[^ ^138^ ^]^ They further demonstrate that the thermal management issues of HEMTs could be addressed through utilizing substrates with high thermal conductivity. In 2022, Kim et al. fabricated transferable surface acoustic wave (SAW) sensors by growing GaN membranes on 2D materials via remote epitaxy.^[^ ^29^ ^]^ The fabricated SAW sensors utilized piezoelectric effects to realize multimodal sensing of pulses, sweat, and UV, and the measured signals were wirelessly transmitted through antennas (Figure 13i). The ultrahigh mechanical coupling coefficient of GaN membranes enables efficient sensing, which substantiates the impact of 2DLT on novel hetero‐integrated platforms.

Thus, while early studies on WBG/2D heterostructure‐based applications primarily relied on transfer methods, the recent advancements on direct growth of WBG materials and 2D layers opened new application fields previously inaccessible. This also offered breakthroughs to overcome the challenges associated with WBG‐based devices (**Table** **2**). Despite these advancements with proof‐of‐concept device demonstrations, many challenges still need to be addressed before WBG/2D heterostructuring technology can be effectively applied in high‐power, high‐frequency electronics, and quantum applications. To name a few, achieving sufficiently high material quality, uniformity, scalability, and fabrication compatibility are the key requirements. The most crucial requisite is materials engineering and optimization by establishing advanced growth processes. Therefore, with the ongoing advancements in WBG/2D heterostructure fabrication techniques, a broader range of device structures and high‐performance devices are expected to be developed. These advances will provide the foundation for enhanced functionality and improved performance in various technological applications.

**Table 2:** Summary of representative studies on WBG/2D heterostructure‐based applications.

Category	Device	WBGs	2D materials	Fabrication technique	Note	Refs.
WBG/2D heterojunction electronic devices	MISFET	β‐Ga_2_O_3_	hBN	dry transfer	Higher threshold voltage and on/off ratio compared to SiO_2_ dielectric	[112]
	JFET	β‐Ga_2_O_3_	WSe_2_	dry transfer	Low subthreshold swing and high on/off ratio	[113]
	Vertical barristor	β‐Ga_2_O_3_	graphene	dry transfer	High‐breakdown electric field	[114]
	Esaki diode	GaN	MoS_2_	Mo film sulfurization	NDR signal in forward bias region	[75]
WBG/2D heterojunction LEDs & photodetectors	LEDs	GaN	MoSe_2_	CVD	Large‐scale heterojunction LED	[115]
	UV & DUV detector	β‐Ga_2_O_3_	graphene	wet transfer	Performance varies depending on the materials and device structures	[116]
		GaN	graphene	wet transfer		[117]
		β‐Ga_2_O_3_	graphene	dry transfer		[118]
	Dual & wide‐range photodetector	ZnO	WSe_2_	dry transfer	Dual, wide‐range photodetectors utilizing two materials with different bandgaps	[73]
		GaN	MoS_2_	dry transfer		[122]
		GaN	MoS_2_	dry transfer		[123]
		GaN	MoSe_2_	dry transfer		[124]
Novel device concepts	LJFET	ZnO	WSe_2_	dry transfer	The on/off operation of the ZnO channel was controlled by photo‐exited charges in the gate layer (WSe_2_)	[125]
	Nano‐generator	ZnO	graphene	hydrothermal	Piezo‐electric ZnO nanorods were grown on defects in the graphene layer	[126]
	Piezo‐photonic diode	ZnO	MoS_2_	dry transfer	The photocurrent of the diode increased when pressure applied	[127]
Performance improvement	HEMT	GaN	graphene	wet transfer	Graphene is utilized as a heat spreader layer in GaN HEMT	[26]
	MQW LED	III‐N	graphene	qvdW epitaxy	Using a graphene template, AlN and AlGaN MQW with less strain and low dislocation density were grown	[130]
Applications via 2DLT	Transferable, flexible LEDs	GaN, ZnO	graphene	pinhole epitaxy	Interfacial 2D layers enable the transfer of III‐N layers to arbitrary substrates, including other III‐V layers and flexible PET film.	[28]
		III‐N, ZnO	graphene	pinhole epitaxy		[133]
		III‐N	hBN	qvdW epitaxy		[134, 135, 136]
	Micro‐LEDs	III‐V	graphene and hBN	remote & qvdW epitaxy		[137]
	HEMT	III‐N	hBN	qvdW epitaxy	AlGaN/GaN HEMT transferred onto heat spreading layer	[27, 138]
	Wearable electronics	GaN	amorphous graphene and BN	remote epitaxy	GaN SAW‐based electronic skin exhibits biomedical sensing performance with high sensitivity and long‐term stability.	[29]

## 5 Summary and Perspectives

In summary, significant efforts have focused on the heterogeneous integration of WBGs with 2D materials, driven by new functionalities that merge these materials. Considering integrating these distinctive materials is not straightforward, various fabrication techniques have been developed to form WBG/2D heterostructures with high material quality and desirable properties. Based on such efforts, numerous applications have been demonstrated, demonstrating the potential and versatility of WBG/2D heterostructures.

The formation of WBG/2D heterostructures was investigated using both transfer‐based approaches and growth‐based approaches. Although the transfer process is simple and can be used to form virtually any kind of 2D materials on WBGs, transfer‐process‐related damage, interfacial contamination, and limited scalability pose significant challenges to maturing the technology. However, whether these challenges will be fully addressed in the foreseeable future is unclear. In contrast, direct growth of 2D materials on WBGs or WBGs on 2D materials is more scalable, repeatable, and reliable, despite the delicacy of the process. Although the available combinations of materials are limited, the direct growth of single‐crystal 2D materials on WBGs is now feasible. Novel growth techniques, such as qvdW epitaxy and remote epitaxy, have enabled the growth of WBGs on 2D materials, and the surface properties of 2D materials have facilitated the reduction of dislocations in WBGs. 2D materials also allow the exfoliation and transfer of WBG layers via 2DLT for heterogeneous integration. Despite these remarkable advances in direct‐growth techniques, obtaining high‐quality WBG/2D heterostructures remains challenging. One of the challenges arises from the harsh growth environment, which can easily damage the interface or surface of the growth templates. In addition, controlling nucleation is also challenging owing to a) the mismatch of lattice properties between WBGs and 2D materials, b) the distinct diffusion behaviors of adatoms and precursors in (quasi‐) vdW epitaxy, and c) the attenuated interaction between adatoms and substrates in remote epitaxy. Thus, growth environments must be tailored specifically to these unique material systems to advance the available material library and the quality of WBG/2D heterostructures.

From an application perspective, WBG/2D heterostructures provide new degrees of freedom for the design of materials and devices. For example, inserting 2D materials into WBG devices enhanced the performance of WBG devices in diverse aspects, including electrical, optical, thermal, and mechanical properties engineered by WBG/2D heterostructures. The growth of high‐quality WBGs with reduced defects on 2D layers has also helped improve device performance. Using 2DLT enabled the transfer of WBG epilayers to various substrates, thereby enabling novel concepts of hetero‐integrated electronic and optoelectronic platforms. However, most reported WBG/2D heterostructure‐based devices show only marginal performance improvements or even poorer performance than their conventional counterparts, inconsistent with theories projecting significant performance enhancements. The unsatisfactory performance stems from imperfect materials and interfaces, underscoring the importance of the growth and integration processes. Developing entirely new device concepts that can fully exploit the distinctive properties of both materials is also crucial to unlocking their full potential, potentially leading to significant advancements in device and functionality.

Moreover, numerous combinations of WBG/2D heterostructures remain unexplored. Given the impracticality of experimentally investigating all these potential combinations, establishing well‐founded theoretical frameworks based on existing research findings is essential. Additionally, developing predictive models using machine learning can significantly enhance our understanding and enable the efficient identification of promising WBG/2D heterostructures, considering these models can predict the material behavior and device performance, guide experimental efforts, and accelerate the discovery of new heterostructures for studying fundamental science, thus contributing to real‐world applications. Hence, combining theoretical insights, novel device structure, advanced synthesis techniques and fabrication processes is expected to unlock the full potential of WBG/2D heterostructures.

## Conflict of Interest

The authors declare no conflict of interest.
